# Effects of an open-label placebo on divergent thinking performance

**DOI:** 10.3389/fpsyg.2026.1833541

**Published:** 2026-07-16

**Authors:** Marlies Stopper, Albert Wabnegger, Janika Saretzki, Corina Scheer, Anne Schienle

**Affiliations:** 1 Section of Clinical Psychology, Department of Psychology, University of Graz, Graz, Austria; 2 Creative Cognition Lab, Department of Psychology, University of Graz, Graz, Austria; 3 Section of Psychological Methods and Assessment, Department of Psychology, LMU, Munich, Germany

**Keywords:** alternative uses, creativity, divergent thinking, performance, mood, open-label/nondeceptive placebos, placebos

## Abstract

**Background:**

Placebos deceptively labeled as “creativity enhancers” have been shown to improve divergent thinking (DT) performance. This study examined whether nondeceptive placebos administered with full transparency, which are also known as open-label placebos (OLPs) can produce comparable effects when framed as enhancing either cognitive or affective processes.

**Methods:**

Participants were randomly assigned to one of three groups that completed the Alternative Uses Task (AUT) twice within a single testing session. After the first AUT (baseline), two groups received a placebo pill, which was introduced either as a “creativity enhancer” or a “mood enhancer”, whereas the third group received no treatment. Objective outcomes included DT performance (fluency, originality, and flexibility); subjective measures comprised ratings of mood, attention, performance perception, and expected/perceived placebo efficacy.

**Results:**

The study included 105 participants (mean age: 29.6 years, SD = 9.63, 62% female). Placebo administration improved subjective outcomes but did not affect DT performance (all *p* > 0.05). It increased self-reported mood (ΔM = 0.36, 95% CI [0.13, 0.58], SE = 0.11) and attention (ΔM = 0.56, 95% CI [0.27, 0.84], SE = 0.14), regardless of labeling condition. Perceived placebo efficacy after the AUT decreased relative to expected efficacy before the experiment (ΔM = −0.66, 95% CI [−1.07, −0.25], SE = 0.21).

**Conclusion:**

These placebo effects were limited to subjective states and were comparable across the creativity- and mood-enhancement suggestion conditions, highlighting the need for future research to further investigate the role of content-specific suggestions in placebo responsiveness.

## 1 Introduction

Divergent thinking (DT) encompasses the ability, the skill, and the process to generate original solutions to open-ended problems by integrating different forms of information in novel ways (Guilford, 1967). Key indicators of DT performance include originality (the uniqueness and novelty of ideas), fluency (the number of ideas), and flexibility (the ability to shift between different categories). DT, combined with convergent thinking - the process of refining and selecting the best ideas into meaningful and/or useful solutions - constitutes the essence of creativity.

Higher DT performance is linked to various positive outcomes, including psychological well-being (Fusi et al., 2024), self-efficacy (Zuo et al., 2021), and resilience (De Lorenzo et al., 2024). Therefore, identifying and implementing strategies to enhance DT is a valuable pursuit.

Two previous studies have demonstrated that placebos (inert substances/treatments) accompanied by positive verbal suggestions, can improve divergent thinking (DT). In one study, Rozenkrantz et al. (2017) found that participants performed better on two DT tasks after receiving a placebo in the form of an odorant described as a creativity enhancer. Similarly, Susnea and Susnea (2020) reported placebo-induced improvements in a DT task, in which the placebo manipulation consisted of written feedback suggesting that participants were more creative than average.

Despite these promising findings, it remains uncertain whether comparable effects can be attained through the use of open-label placebos (OLPs), which are administered with full disclosure of their inert nature. Notably, some studies suggest that OLPs may yield effects comparable to deceptive placebos (DPs) in certain contexts (e.g., Disley et al., 2021; Stopper et al., 2024). Given their ethical advantages, including respect for autonomy and informed consent (Annoni, 2018), OLPs have emerged as a preferred approach within placebo research.

For the present study, two types of OLPs were developed based on prior research in creativity and placebo effects. On the one hand, studies have shown that placebos administered as ‘mental booster’ can improve cognitive performance. For example, Parker et al. (2011) found that a placebo described as a memory aid enhanced prospective memory performance. Similarly, Oken et al. (2008) demonstrated that an inert pill labeled as a ‘cognitive enhancer’ improved various functions, including working memory, selective attention, and inhibitory control, in healthy older adults. However, other studies have also found that placebos presented as cognitive neuroenhancers had no effect on performance (e.g., Schwarz and Büchel, 2015; Winkler and Hermann, 2019).

On the other hand, placebos—both deceptive and nondeceptive—have been shown to influence emotional processes. They can alleviate negative emotional states, such as anxiety, sadness, disgust (Petrovic et al., 2005; Schienle et al., 2022) and enhance mood (Baker et al., 2022). The latter aspect is particularly relevant to creativity, as inducing a positive mood has been found to improve divergent thinking performance. In a study by Yamada and Nagai (2015), participants in the experimental group listened to “happy music” and reflected on joyful experiences to induce a positive mood, while a control group received no mood induction. Results indicated that the positive mood condition led to improved DT performance. This finding aligns with the broaden-and-build theory by Fredrickson (2001), which suggests that positive emotions expand cognitive flexibility and creativity.

Based on the presented findings, this exploratory study aimed to examine the effects of OLPs on DT performance in healthy adults using the Alternative Uses Task (AUT; Wallach and Kogan, 1965). Participants were randomly assigned to one of three groups: two placebo groups and a control group that received no placebo. To investigate the most effective strategy for administering OLPs to enhance DT, the verbal suggestion accompanying the placebo was systematically varied. One placebo group received a pill introduced as a “creativity enhancer,” while the other was told they were receiving a “mood enhancer.” We assessed both objective outcome measures (DT performance regarding fluency, originality, and flexibility), as well as subjective measures (ratings of mood, attention, expected and perceived DT performance, expected and perceived placebo efficacy). Participants completed the AUT twice, before and after placebo administration, with the initial assessment serving as a baseline measure of performance.

## 2 Methods

### 2.1 Participants

Participants were recruited between February and December 2025 through social media, email distribution lists, and postings on the university campus. Psychology students from the University of Graz received course credit for their participation, whereas other participants did not receive any reimbursement.

Participants were allocated to one of three groups in a 1:1:1 ratio (*n* = 35 per group) using a computer-generated randomization procedure. The allocation sequence was generated using an online random number generator^1^ and applied to assign participants upon enrolment. Two groups received an OLP in pill form either labeled as a creativity enhancer or mood enhancer, while the control group received no placebo treatment.

The inclusion criteria required participants to be aged 18 years or older. Exclusion criteria were self-reported mental disorders or medication causing impaired cognitive function, as well as poor German language skills (language level below C1). An online pre-screening was conducted to control whether the inclusion criteria were met.

The sample size had been determined via G*Power (Version 3.1.9.7; Faul et al., 2007). For a small effect size of *f* = 0.15, with an alpha error probability of 0.05 and a power of 0.95, a sample size of 93 participants was required. In order to prepare for potential dropouts during the testing or analysis phase, we aimed for a sample size of 105 subjects. No drop-outs occurred. The study was approved by the ethics committee of the University of Graz (GZ. 39/38/63 ex 2024/25) and was conducted in accordance with the Declaration of Helsinki. The study was preregistered on the German Register for Clinical Studies (DRKS00035385).

### 2.2 Design

This study was a randomized controlled trial with a between-subjects group factor and a within-subject pre–post (baseline vs. post-intervention) design. Participants were randomly assigned to one of three groups: the creativity enhancer (CE) group, the mood enhancer (ME) group or the control group. All participants first completed an initial AUT to establish baseline DT performance. The second AUT was conducted after administering the placebo to the intervention groups.

### 2.3 Procedure

**Figure 1:**
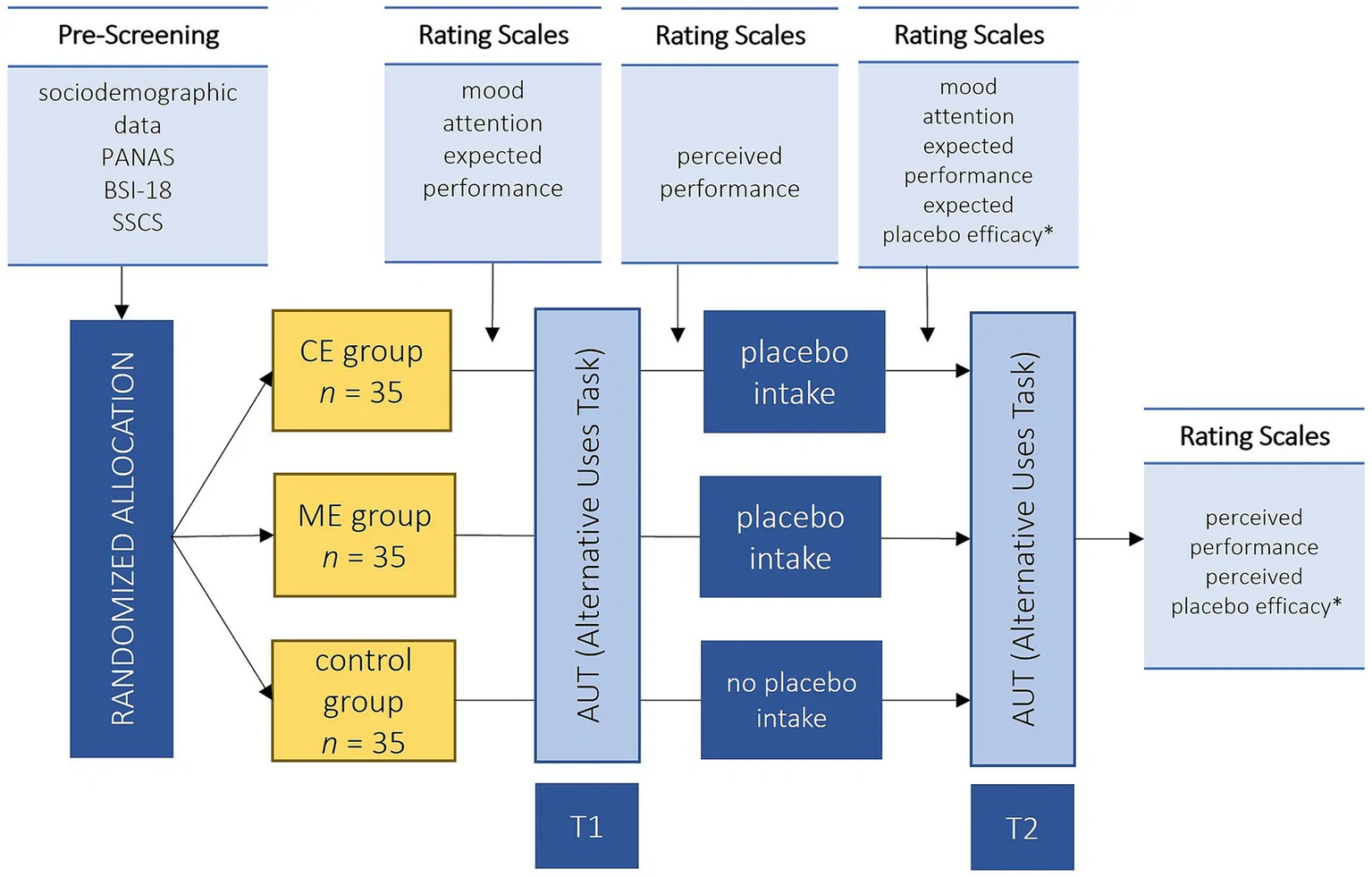
Testing procedure. CE, creativity enhancer; ME, mood enhancer; PANAS, positive and negative affect schedule; BSI-18, brief symptom inventory – short version; SSCS, short scale of creative self. *Ratings for expected and perceived placebo efficacy were only assessed among the CE and ME group.

The study comprised two sections: an online survey conducted via LimeSurvey and a subsequent testing in the lab (Figure 1). The online survey served as a preliminary screening instrument (for inclusion/exclusion criteria), assessed sociodemographic data and included the questionnaires (2.5). Subjects who met the inclusion criteria were then contacted by e-mail and invited to the testing session.

Participants were randomly allocated to one of the three groups and received a detailed information sheet outlining the testing procedure. After consenting, they were then asked to take a seat in front of a computer on which the AUT’s procedure was briefly explained.

All three groups completed the AUT twice. Prior to the second AUT, the two intervention groups received the placebo. To ensure standardization, the placebo instructions were delivered in written form on the computer screen. The instruction for both groups included: (1) an explanation regarding the true nature of the true nature of the placebo (“You will now be given a nondeceptive placebo, which is a capsule filled with potato starch.”), (2) information regarding the expected placebo effect (creativity enhancer: “The placebo is intended to help boost your creativity.” vs. mood enhancer: “The placebo is intended to help improve your mood.”), and (3) references to studies reporting the positive impact of placebos on creativity or mood. The experimenter subsequently reiterated the instructions verbally and asked whether they had been fully understood, providing additional clarification if required. In particular, if participants requested clarification regarding the placebo’s potential effects, the experimenter explained that any effects would arise from placebo mechanisms rather than from pharmacological properties of the capsule itself. Participants were then administered the placebo and instructed to pause for 1 minute and contemplate on the placebo’s expected effect. The one-minute interval was introduced to allow a brief period for expectancy consolidation (“This helps me to boost my mood/creativity”) as the formation and stabilization of expectations is considered a key mechanism underlying open-label placebo effects (e.g., Buergler et al., 2023). The second AUT was administered immediately thereafter within the same laboratory session. Due to the nature of the intervention, neither participants nor the experimenter were blinded to group allocation.

### 2.4 Placebo

Vegan hard capsules containing potato starch were used as the placebo. They were filled into a small, white pill container with the label “placebo” on it. Based on the assigned placebo group, an additional inscription on the label either read “to increase creativity” or “to improve mood” (Supplementary Figure S1). Subjects were instructed to take one capsule out of the container and swallow it with a sip of water provided in a drinking cup.

### 2.5 Alternative uses task (AUT)

The Alternative Uses Task (AUT; Wallach and Kogan, 1965) is the most common DT task in creativity research (Saretzki et al., 2024) and asks participants to generate creative uses for everyday objects. In the current study, the AUT used two sets (set A: sock, pencil, purse; set B: pillow, comb, belt) containing different but comparable objects in terms of shape, size and material.

Subjects were shown the name of an object on a computer screen, followed by the instruction: “Please try to list as many alternative and creative uses for this object as possible, where “creative” refers to ideas that are unusual, original, innovative, or simply different.” Each object was presented with a time limit of 2 minutes. Participants typed their responses directly into a text box on the computer. To ensure the task was understood correctly, an example demonstration (object: paperclip; possible alternative uses: earring, fishhook, bookmark, etc.) was provided at the start of the task. Subjects completed the AUT twice. The order of the sets and the objects within each set was randomized.

#### 2.5.1 AUT scoring

For AUT scoring, fluency, originality and flexibility were assessed; repetitive, incomplete and senseless responses were discarded.

Fluency: Fluency relates to the number of responses generated by an individual. Fluency scores were assessed by calculating the average number of alternative uses for each set. Participants’ responses were counted and averaged independently by two trained raters. In this regard, no discrepancies were found.

Originality: Originality refers to the novelty and innovation of each response. In this context, responses were evaluated using OSCAI (Open Creativity Scoring with Artificial Intelligence; Organisciak et al., 2023), an automated scoring system based on Large Language Models (LLMs), and rated on a scale from 1 (very unoriginal) to 5 (very original). For response aggregation, a max-3-scoring was employed, where the average of the three most original responses for each object was calculated. If less than three responses were given, the evaluations of available responses were averaged (e.g., Saretzki et al., 2025).

Flexibility: Flexibility refers to the number of different categories an individual’s ideas span (e.g., play/entertainment, decoration/ornament, weapon/harm). In this regard, the two previously mentioned raters counted the number of categories for each object. To control for interrater-reliability, an intraclass correlation coefficient (ICC) was calculated, which exhibited excellent reliability (ICC(3,2) = 0.996, 95% CI: [0.995, 0.998]; Supplementary Table S1). Flexibility scores were assessed by averaging the number of categories for each set.

### 2.6 Questionnaires

#### 2.6.1 Positive and negative affect schedule (PANAS)

To examine trait affect, the Positive and Negative Affect Schedule (PANAS; Breyer and Bluemke, 2016) was administered to the participants. The PANAS is a questionnaire consisting of 20 items describing various negative and positive emotions. In the current study, subjects were asked to indicate the extent to which they have experienced each feeling within the last year on a scale from 1 (not at all) to 5 (very strongly). For evaluation, a positive and negative affect score (Cronbach’s alpha: positive: 0.86, negative: 0.81) was calculated (Table 1).

**Table 1:** Group characteristics (means, ± standard deviations).

	Creativity enhancer *n* = 35	Mood enhancer *n* = 35	Control (no placebo) *n* = 35
Sex ratio (male/female/diverse)	13/22/0	16/18/1	9/25/1
Mean age (years)	30.00 ± 9.48	29.34 ± 9.71	29.37 ± 9.99
PANAS positive affect*	3.53 ± 0.62	3.45 ± 0.63	3.57 ± 0.56
PANAS negative affect*	2.47 ± 0.54	2.27 ± 0.59	2.21 ± 0.58
BSI-18 total (GSI)**	11.0 ± 6.75	11.0 ± 8.95	9.91 ± 6.41
SSCS total (creative self-concept) ^†^	3.69 ± 0.77	3.59 ± 0.69	3.68 ± 0.87

PANAS, positive and negative affect schedule, BSI-18, brief symptom inventory – short version, GSI, global severity index; SSCS, short scale of creative self.

Item response scales: *1–5, **0–72, ^†^1–5.

#### 2.6.2 Brief symptom inventory (BSI-18)

Given the established correlation between creativity and mental disorders (Zhao et al., 2022), participants were also given the BSI-18 (Brief Symptom Inventory; Franke, 2000) to evaluate psychological distress and psychiatric conditions. Subjects were presented with a list of 18 psychological and physiological symptoms and were required to indicate the extent to which these symptoms have occurred within the past 7 days on a scale from 0 (not at all) to 4 (very strongly). Consequently, the three six-item scales measuring somatization, depression, and anxiety were derived, with no significant differences observed between groups. Therefore, only the Global Severity Index (GSI; Cronbach’s alpha: 0.84), representing the total BSI score, is reported in Table 1.

#### 2.6.3 Short scale of creative self (SSCS)

As people vary in their creative self-concept, participants were also administered the Short Scale of Creative Self (SSCS; Karwowski et al., 2018). The SSCS is an 11-item Likert scale designed to assess individuals’ perceptions of their own creative ability. It comprises two scales: Creative Self-Efficacy (CSE; e.g., Item 4: “I trust my creative abilities”) and Creative Personal Identity (CPI; e.g., Item 1: “I think I am a creative person”). Responses are given on a scale from 1 (disagree) to 5 (agree). For the analysis, a composite score representing overall Creative Self Concept (CSC, Cronbach’s alpha: 0.90) was calculated by averaging the items from both scales (Table 1).

### 2.7 Rating scales

Prior to each AUT, participants were instructed to assess their mood using the valence scale from the Self-Assessment Manikin (SAM; Bradley and Lang, 1994) ranging from 1 (very bad) to 9 (very good), and to evaluate their level of attention on a 10-point likert scale with 1 meaning “feeling hardly attentive” and 10 meaning “feeling very attentive.” Furthermore, subjects were instructed to report their expected task performance (“How well will you perform in the task?”) before and their perceived task performance (“How well have you performed in the task?”) after each AUT completion on a 10-point-likert scale (1 – very bad, 9 – very good).

Before the second AUT, subjects from the intervention groups were also asked on how much they believed that the placebo would help with task performance (expected placebo efficacy) using the previously referenced 10-point likert scale. Perceived placebo efficacy (“How much has the placebo helped with task performance?”) was rated after the task with the same rating scale.

### 2.8 Statistical analysis

Three by two analyses of variance (ANOVAs) were calculated to test the effects of INTERVENTION (between-subjects: Creativity Enhancer Group, Mood Enhancer Group, Control Group) and TIME (within-subjects: before and after intervention) on AUT-scores (fluency, originality, flexibility). Sex, age, PANAS scores (positive affect, negative affect), BSI-18 (GSI), and SSCS total score were initially included as covariates. Sensitivity analyses comparing models with and without covariates yielded comparable results across all outcome variables. Accordingly, the reported ANOVAs are based on models without covariates.

Additionally, further ANOVAs were computed to assess the effects of INTERVENTION and TIME on subjective measurements regarding mood, attention, expected and perceived performance as well as expected and perceived placebo efficacy.

Effects were considered statistically significant when the observed *p*-value was below 0.05. For analysis of (co)variance, effect sizes are reported as *ηp*^2^. Analyses were conducted with JAMOVI (2.7.17) and IBM SPSS Statistics version: 30.0.0.0 (172).

## 3 Results

### 3.1 Descriptive data

The three groups did not differ in mean age (*f*(2,102) = 0.05, *p* = 0.95), gender ratio (*χ*^2^(4) = 4.09, *p* = 0.36), or any of the administered questionnaires (all *p* > 0.05; Table 1).

### 3.2 Objective outcomes

**Figure 2:**
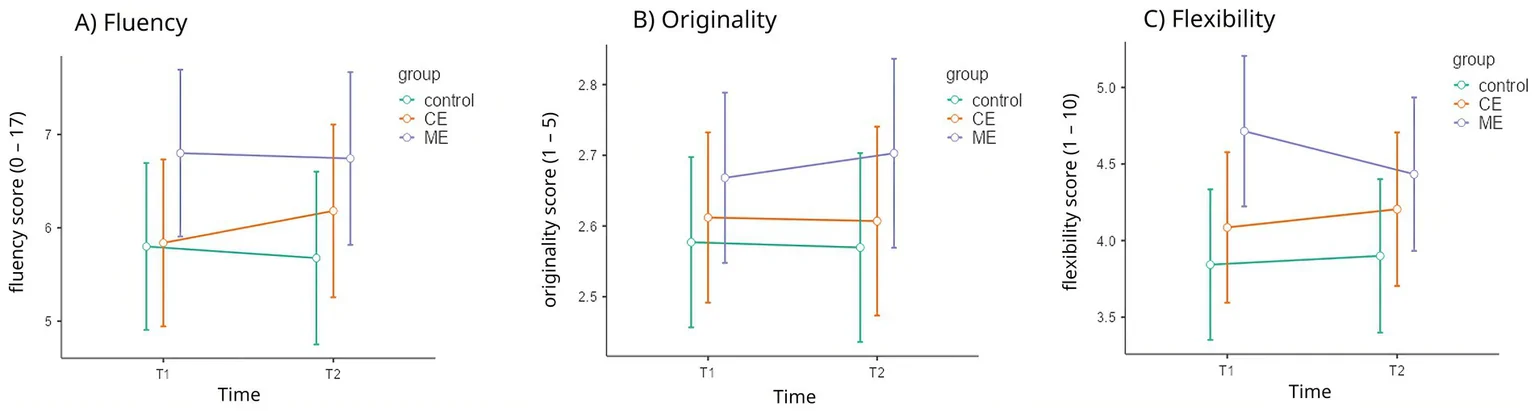
Divergent thinking performance (means and 95% confidence intervals). **(A)** Fluency, **(B)** Originality, and **(C)** Flexibility scores measured at T1 and T2 for the three experimental conditions. CE, creativity enhancer; ME, mood enhancer.

Descriptive statistics for DT performance measures are displayed in Table 2, line charts regarding fluency, originality and flexibility are displayed in Figure 2. Interpretations of significant interaction effects were based on the analysis of the confidence intervals (CIs; Garofalo et al., 2022).

**Table 2:** Divergent thinking performance (means, 95% C-interval [CI]).

	Creativity enhancer group		Mood enhancer group		Control group	
	T1	T2	T1	T2	T1	T2
	*n* = 35		*n* = 35		*n* = 35	
Fluency*	5.84 [4.92–6.75]	6.18 [5.17–7.19]	6.80 [5.80–7.80]	6.74 [5.77–7.72]	5.80 [4.98–6.62]	5.68 [4.82–6.53]
Originality**	2.61 [2.49–2.74]	2.61 [2.46–2.76]	2.67 [2.55–2.79]	2.70 [2.60–2.81]	2.58 [2.45–2.70]	2.57 [2.42–2.72]
Flexibility^†^	4.09 [3.57–4.60]	4.20 [3.65–4.76]	4.71 [4.16–5.27]	4.43 [3.93–4.94]	3.84 [3.41–4.28]	3.90 [3.43–4.37]

*Range = 0–17, **range = 1–5, ^†^range = 1–10; T1 (baseline); T2 (after placebo administration in the placebo groups).

*Fluency*: The interaction between GROUP and TIME (*F*(2,102) = 1.15, *p* = 0.32, *ηp*^2^ = 0.02), as well as the main effects GROUP (*F*(2,102) = 1.46, *p* = 0.24, *ηp*^2^ = 0.03) and TIME (*F*(1,102) = 0.16, *p* = 0.69, *ηp*^2^ < 0.01) did not reach statistical significance.

*Originality*: For originality, the analysis yielded no significant effects for GROUP × TIME (*F*(2,102) = 0.23, *p* = 0.80, *ηp*^2^ < 0.01) GROUP (*F*(2,102) = 0.94, *p* = 0.40, *ηp*^2^ = 0.02) or TIME (*F*(1,102) = 0.07, *p* = 0.80, *ηp*^2^ < 0.01).

*Flexibility:* Similarly, no significant effects were observed for flexibility (GROUP × TIME: (F(2,102) = 1.59, *p* = 0.21, *ηp*^2^ = 0.03); GROUP: *F*(2,102) = 2.27, *p* = 0.11, *ηp*^2^ = 0.04; TIME: (*F*(1,102) = 0.13, *p* = 0.72, *ηp*^2^ < 0.01).

### 3.3 Subjective outcomes

**Figure 3:**
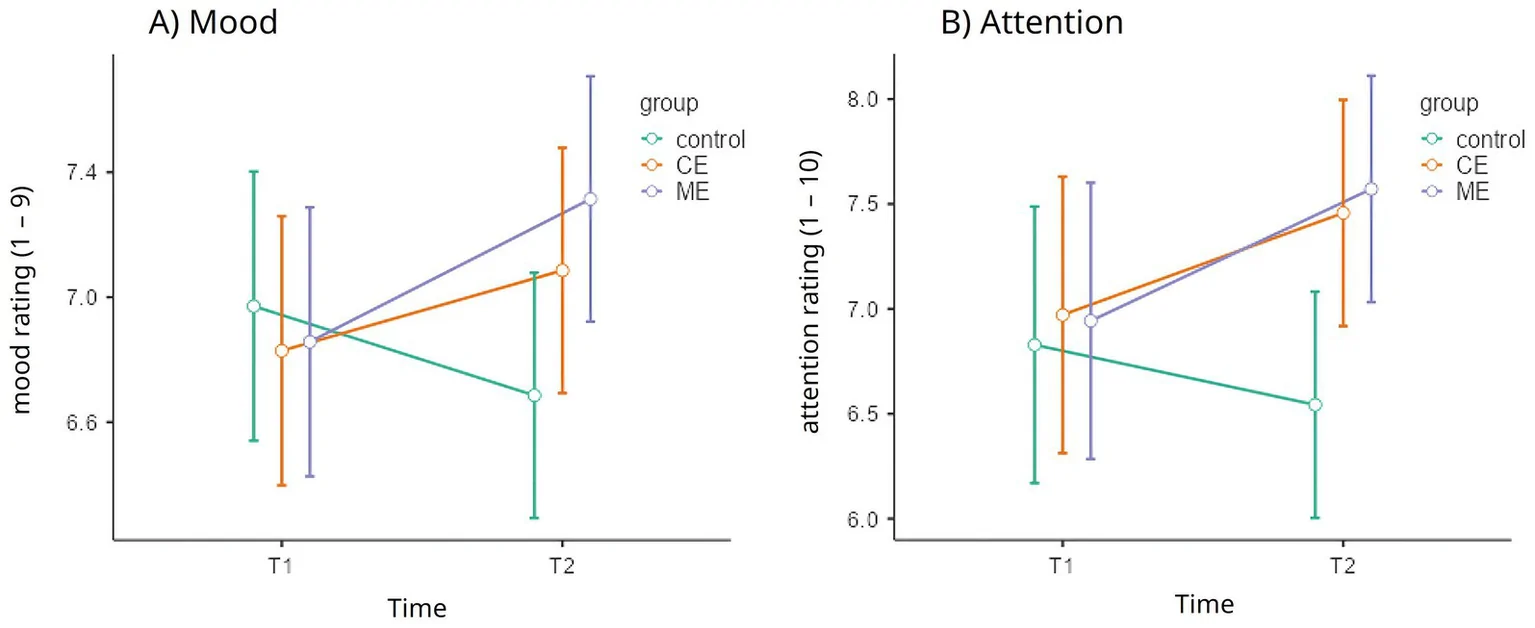
Mood and attention ratings (means and 95% confidence intervals). **(A)** Mood, and **(B)** Attention ratings measured at T1 and T2 for the three experimental conditions. CE, creativity enhancer; ME, mood enhancer.

*Mood*: There was a significant interaction between GROUP and TIME (*F*(2, 102) = 4.53, *p* = 0.01, *ηp*^2^ = 0.08; Figure 3, chart A), while main effects were not significant. When considering change-from-baseline scores, a similar increase in mood was observed in both intervention groups, with overlapping confidence intervals. The CE group showed a small increase (ΔM = 0.26, 95% CI [−0.05, 0.56], SE = 0.15), and the ME group showed a slightly larger increase (ΔM = 0.46, 95% CI [0.11, 0.80], SE = 0.17). In contrast, the control group showed a slight decrease in mood (ΔM = −0.29, 95% CI [−0.72, 0.15], SE = 0.22).

*Attention*: A significant main effect of TIME was observed (*F*(1,102) = 5.30, *p* = 0.02, *ηp*^2^ = 0.05), along with a significant interaction between GROUP and TIME (*F*(2,102) = 5.61, *p* = 0.01, *ηp*^2^ = 0.10; Figure 3, chart B). Both intervention groups perceived an increase in attention: the CE group showed a mean change of ΔM = 0.49, 95% CI [0.08–0.89], SE = 0.20; the ME group showed a mean change of ΔM = 0.63, 95% CI [0.20–1.05], SE = 0.21. In contrast, the control group exhibited a mean change of ΔM = −0.29, 95% CI [−0.72–0.15], SE = 0.22.

**Figure 4:**
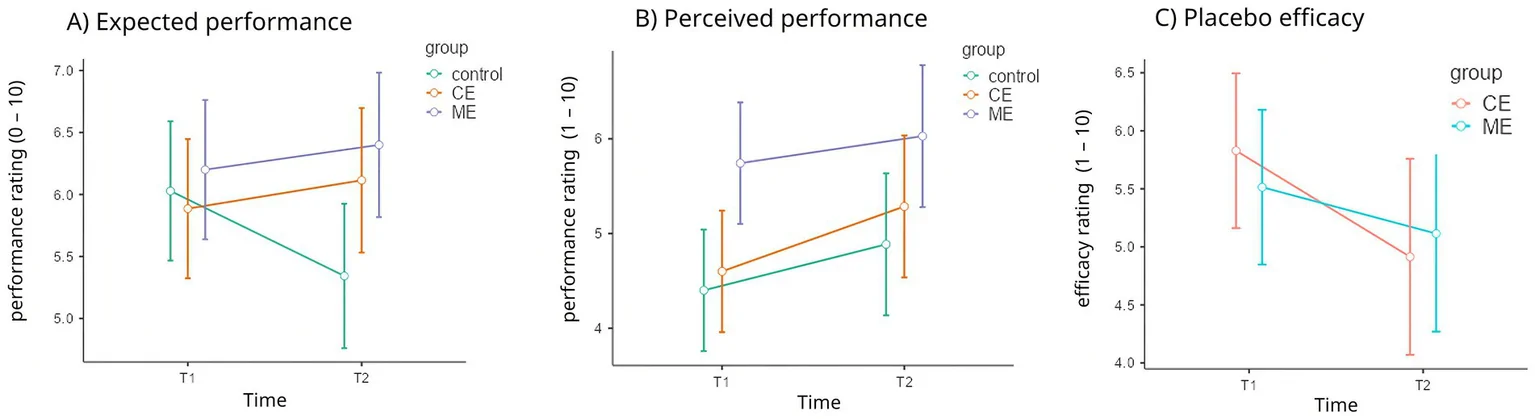
Ratings for subjective performance and placebo efficacy (means and 95% confidence intervals). **(A)** Expected performance, **(B)** Perceived performance, and **(C)** Placebo efficacy ratings measured at T1 and T2 for the three experimental conditions. CE = creativity enhancer; ME = mood enhancer.

*Expected performance*: A significant interaction between GROUP and TIME was observed (*F*(2,102) = 3.64, *p* = 0.03, *ηp*^2^ = 0.07; Figure 4, chart A). Both intervention groups reported an increase in expected performance before the second AUT, with the CE group showing a mean change of ΔM = 0.23, 95% CI [−0.41, 0.87], SE = 0.32, and the ME group showing a mean change of ΔM = 0.20, 95% CI [−0.34, 0.74], SE = 0.27. In contrast, the control group exhibited a decrease in expected performance (ΔM = −0.69, 95% CI [−1.16, −0.22], SE = 0.23).

*Perceived performance*: For perceived performance, analysis revealed a significant main effect GROUP (*F*(2,102) = 4.11, *p* = 0.02, *ηp*^2^ = 0.08) and TIME (*F*(1,102) = 8.27, *p* = 0.01, *ηp^2^* = 0.08; Figure 4, chart B). Regarding the main effect GROUP, participants from the ME group reported the highest perceived performance (M = 5.89, CI [5.25, 6.52], SE = 0.32), followed by the CE group (M = 4.94, CI [4.31, 5.58], SE = 0.32) and control group (M = 4.64, CI [4.01, 5.28], SE = 0.32). Moreover, across all participants, perceived performance increased from T1 to T2 (ΔM = 0.49, 95% CI [0.15, 0.82], SE = 0.17).

*Placebo efficacy*: The analysis of placebo efficacy yielded a significant main effect TIME (*F*(1,68) = 10.32, *p* < 0.001, *ηp*^2^ = 0.13; Figure 4, chart C). Both intervention groups rated the perceived placebo efficacy lower than the expected efficacy with a mean change of ΔM = −0.66, 95% CI [−1.07, −0.25], *SE* = 0.21. The main effect for GROUP and the interaction were not significant.

## 4 Discussion

To our knowledge, the present study is the first to examine the influence of an open-label placebo (OLP) on divergent thinking (DT) performance. Contrary to our hypothesis, placebo administration had no significant effect on participants’ DT performance regarding fluency, originality, or flexibility.

These findings contrast with previous studies on deceptive placebos (DP), which reported improvements in DT performance (Rozenkrantz et al., 2017; Susnea and Susnea, 2020). However, several DP studies investigating other cognitive outcomes have likewise yielded null results. For example, Schwarz and Büchel (2015) demonstrated that placebo-induced expectations did not alter performance in a Flanker task. Similarly, Winkler and Hermann (2019) found that a DP administered as a nasal spray did not affect performance on an attention task, and Kleine-Borgmann et al. (2021) reported that a 21-day DP intervention failed to improve students’ performance on a midterm examination.

In contrast to the absence of effects on objective performance, the present study revealed OLP effects on subjective measures. Placebo intake positively influenced performance expectations and led to improvements in mood and self-reported attention. Notably, however, the placebo did not affect participants’ perceived performance. Previous DP studies have reported a dissociation between performance perception and objective performance (e.g., Schwarz and Büchel, 2015; Blokland, 2023). Very similar to our results, Blokland (2023) showed that placebo recipients felt more motivated and attentive, despite no changes in actual performance on tasks assessing word learning, working memory, Tower of London performance, or spatial pattern separation. Thus, the placebo improved the subjective state without translating into objective cognitive benefits. This finding aligns with existing literature. A recent meta-analysis by Fendel et al. (2025) demonstrated that nondeceptive placebos can significantly improve symptoms compared to no-treatment controls or standard care, especially concerning subjective outcomes. Conversely, no consistent effects were noted for objective or physiological outcomes.

In the present study, all participants - independent of group assignment -reported higher performance in the second AUT. This likely reflects concepts about practice effects and increased task familiarity, which are commonly assumed to enhance performance. However, this perceived improvement was not supported by the objective data.

An innovative aspect of the present study was the use of different placebo labels and verbal suggestions. The OLP was framed either as a “creativity enhancer” or as a “mood enhancer.” Surprisingly, these suggestions did not produce differential effects on either objective or subjective outcome measures. Both placebo types similarly improved participants’ subjective state, as reflected in higher self-reported mood and attention. One possible explanation is that both suggestions may have created overlapping expectations. Both instructions related to positively valued, preferred states (being happier/more creative) and may not have been strong enough to yield separate profiles. Participants might have merely inferred that “things will improve with the OLP.” Another explanation could be that placebo effects do not solely depend on specific outcome expectations but also on broader contextual and ritualistic aspects of treatment (Miller and Kaptchuk, 2008). The mere act of receiving and ingesting a pill may already function as a powerful cue that signals care, intervention, or change, thereby eliciting nonspecific improvements in subjective states such as mood or alertness.

Another possibility is that the placebo intervention primarily induced a general anticipatory state rather than a domain-specific expectation. Being told that the placebo is going to be a helping agent could increase overall motivation, which in turn might translate into improved mood and perceived attentiveness. Such nonspecific expectancy or anticipatory mechanisms may operate independently of the precise verbal framing of the placebo.

Regarding placebo efficacy, both intervention groups rated the placebo as slightly less effective than anticipated. Similar findings were reported by Schienle et al. (2022), where participants indicated that the OLP did not meet expectations. This phenomenon reflects expectancy violation, in which discrepancies between expected and actual outcomes diminish placebo effects (Colloca et al., 2019). In the present study, it is plausible that expectations regarding the placebo’s efficacy were disproportionately high relative to the modest and subtle effects typically associated with OLP on objective outcomes (SMD = 0.09; Fendel et al., 2025).

### 4.1 Limitations and future directions

First, although expected efficacy ratings were within the average range, participants may have been skeptical about the plausibility of OLPs enhancing DT (Haas et al., 2021; Locher et al., 2021). Such skepticism may have contributed to the lack of objective changes, and alternative placebo modalities might be more effective. For instance, Rozenkrantz et al. (2017) reported a significant placebo effect on DT using an odorant, which likely produces a more immediate and perceptible effect compared with capsules that require digestion. Since plausibility was not evaluated, this constitutes a limitation of the present study.

Second, it is possible that practice-related expectations may have confounded placebo-related expectations. Following the completion of the testing phase, several participants reported that performing the AUT for the second time felt easier due to their familiarity with the task. For example, one participant stated, “The second time I performed the task, I already knew what to do.” This likely accounts for the higher subjective performance ratings observed during the second administration across all three groups.

Third, unlike pain or emotional states, which are accompanied by bodily sensations that facilitate their detection, changes in cognitive processes may be less perceptible. This reduced tangibility could explain previously mentioned disappointment effects; i.e.: if placebo-induced changes go unnoticed, individuals may perceive the placebo as ineffective. However, research indicates that the perception of sensations targeted by placebo administration is crucial, and that attentional focus on these sensations can even enhance placebo responses (e.g., Geers et al., 2006; Barbiani et al., 2024).

Fourth, our findings are limited to healthy adults who exhibited above-average ratings in creative self-concept and mood. Interventions aimed at enhancing cognitive processes such as DT may prove more effective in populations with lower baseline performance and more negative mood states, where the potential benefits of treatment success are more pronounced.

Fifth, this study employed a single-dose placebo administration. Given the complexity of DT, which relies on the interaction of multiple cognitive components - including semantic retrieval, inhibition of automatic responses, and flexible shifts in attentional focus (Palmiero et al., 2022) - a longer duration of placebo intake may be necessary to induce measurable positive effects. Therefore, future research should investigate the impact of repeated placebo administration.

Another limitation of the present study is that creative cognitive potential was assessed exclusively using the AUT. The AUT (Guilford, 1967; Torrance, 1972) is the most frequently used DT task (Saretzki et al., 2024). However, more realistic DT tasks, including more complex scenarios such as social, emotional, scientific, or business problems that are sometimes adapted to the background of the sample (e.g., business students; Mumford et al., 1998; Okuda et al., 1991; Reiter-Palmon et al., 2009; Runco et al., 2016) appear to be increasingly used, and may capture additional aspects of creative potential that are not fully reflected in the AUT.

## 5 Conclusion

The present study found no evidence that an OLP enhances DT performance. However, it did improve participants’ subjective state, reflected in higher self-reported mood and attention. Moreover, the absence of differential effects between the creativity enhancer and mood enhancer labels suggests that the two framings may have elicited overlapping expectations. Alternatively, the observed effects might also be attributed to broader contextual and ritualistic aspects of the treatment. The results further highlight the importance of considering expectancy violations and the limited perceptibility of cognitive changes when evaluating placebo efficacy.

## Data Availability

The raw data supporting the conclusions of this article will be made available by the authors, without undue reservation.

## Footnotes

1
https://www.gigacalculator.com/randomizers/randomizer.php
