# Supplementary material for: Effects of an open-label placebo on divergent thinking performance
Source: Front Psychol. 2026 Jul 16;17:1833541. doi: 10.3389/fpsyg.2026.1833541 (PMC13422469; doi:10.3389/fpsyg.2026.1833541)
Supplement: Supplementary file 1 [file Table_1.docx]

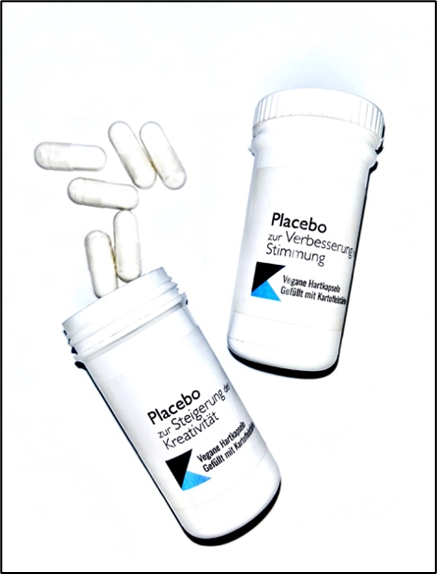
Supplementary Material

**Figure S1.** Pill container with different labels (german): “Placebo to increase creativity“ vs. “Placebo to improve mood“

| Level | ME Group | CE Group | Control Group |
| --- | --- | --- | --- |
| Item_1_ | .981 [.962, .990] | .988 [.976, .994] | .985 [.970, .992] |
| Item_2_ | .986 [.973, .993] | .988 [.976, .994] | .992 [.984, .996] |
| Item_3_ | .992 [.984, .996] | .984 [.969, .992] | .994 [.987, .997] |
| Item_4_ | .989 [.978, 994] | .991 [.982, .995] | .994 [.988, .997] |
| Item_5_ | .990 [.979, .995] | .990 [.980, .994] | .992 [.985, .996] |
| Item_6_ | .973 [.947, .986] | .989 [.977, .994] | .989 [.979, .995] |
| Total | .995 [.991, .998] | .997 [.993, .998] | .997 [.995, .999] |

Item_1_ = Sock. Item_2_ = Pencil. Item_3_ = Wallet. Item_4_ = Belt. Item_5_ = Comb. Item_6_ = Pillow.

**Overall:** ICC(3,2) = **.996,** 95% CI: [.995, .998]

**Table S1.** ICC3k: DT flexibility ratings performed by two trained raters

ME = mood enhancer, CE = creativity enhancer
